# Human cord blood (hCB)-CD34^+^ humanized mice fail to reject human acute myeloid leukemia cells

**DOI:** 10.1371/journal.pone.0217345

**Published:** 2019-09-19

**Authors:** Olga Tanaskovic, Maria Vittoria Verga Falzacappa, Pier Giuseppe Pelicci

**Affiliations:** 1 Department of Experimental Oncology at the IEO, European Institute of Oncology IRCCS, Milan, Italy; 2 Department of Oncology and Hemato-Oncology, University of Milan, Milan, Italy; Purdue University, UNITED STATES

## Abstract

Since their appearance, humanized mice carrying human immune system seemed promising tools to study the crosstalk between cancer and immunity. The NOD-*scid*IL2Rgamma^null^ (NSG) mice engrafted with human cord blood (hCB)-CD34^+^ cells have been proposed to be a valuable tool to reproduce human immune system in mouse. However, the lack of solid evidences on the functionality of their human immune components limits their usage in immune-oncology. We report that (hCB)-CD34^+^ cells lose their ability to propagate and originate bone marrow-derived human immune cells after two serial transplantations in NSG mice. We demonstrate that transplants of bone marrow patient-derived acute myeloid leukemias (hAMLs) grow very similarly in the humanized (hCB)-CD34^+^ NSG and parental NSG mice. The similar extent of engraftment and development of leukemias in (hCB)-CD34^+^ NSG and controls suggests a poor human immune response against not compatible hAMLs. Our findings suggest that (hCB)-CD34^+^ NSG mice are transient and/or incomplete carriers of the human immune system and, therefore, represent a suboptimal tool to study the interaction between tumor and immune cells.

## Introduction

*In vitro* studies are excellent tools to investigate pathways and interactions among few cellular and molecular components, however they do not allow to fully recapitulate complex biological mechanisms that require several cellular and molecular players. For this reason, they are incomplete models to explore the role of the immune system in many complex physiological and pathological conditions. The immune-oncology field aims to study, exploit and manipulate the immunity to recognize and eliminate cancer cells. In order to model the interactions between the human immune system and the human tumoral components, it is necessary to reproduce both in an *in vivo* context. Human xenografted tumors are well established *in vivo* model systems, where human cancer cells or tumor biopsies are heterotransplanted into immunodeficient rodents. On the other hand, humanized mice, which carry a human immune system, have been developed over the past three decades[1,2]. The development of humanized mouse models relies on the use of immuno-deficient mice, which allow the engraftment of human hematopoietic stem cells (hHSCs) and tissues, followed by generation of a functional human immunity in the murine context[3]. Over the past years, different immune-deficient mouse strains have been developed and tested for hHSC and tissue engraftment. Many steps forward have been made to obtain a good level of engraftment of the human cells. Initially, CB17-scid mice were shown to support the engraftment of hHSCs only at low level, due to the presence of murine immune barriers (i.e. NK cells)[4]. Consecutively, NOD-scid mice allowed good engraftment of hHSCs, but their relatively short life span and residual innate immunity represented considerable limitations[5]. Finally, NOD-*scid*IL2Rgamma^null^ (NSG) mice, which lack all the components of murine innate immunity and allow a highly efficient engraftment of human HSCs[6,7], became a gold standard to recapitulate the human immune system in mice[8].

In principle, the development of a complete human immune system in NSG mouse models can be achieved by transplantation of human peripheral blood leukocytes[9], human stem cells (originated from peripheral blood, cord blood or bone marrow) or bone marrow (BM), fetal liver and thymus along with autologous fetal liver HSCs[6]. However, recapitulation of human immunity in NSG mice is often followed by the development of graft-versus-host disease (GvHD)[10] or non-functional human T cell compartment in these mice[11]. Despite their caveats, humanized NSG mouse models were proposed to be an essential tool to study infectious diseases, autoimmunity and drug metabolism, due to a partially functional human immune system. However, very few reports in cancer research took advantage of this model[12–14] and the worth of their usage in immune-oncology remained controversial until now.

## Material and methods

### Mice

NOD-*scid*IL2Rgamma^null^ (NSG) mice were provided from breeding area of the mouse facility at European Institute of Oncology. Animals were housed in the animal facility at European Institute of Oncology, in pathogen-free conditions. All the procedures related to mouse use have been communicated and approved by the Italian Ministry of Health (Project number 15/2016 PR, approved on 12/01/2016).

### Humanization of NSG mice and propagation of (hCB)-CD34^+^ cells

(hCB)-CD34^+^ cells were purchased from Stem Cell Technologies (Cat. No 70008). The cells originate from human cord blood of mixed healthy donors. For NSG humanization, 400.000 cells per mouse were transplanted in sub-lethally irradiated (1 Gy), 6-8 weeks old NSG mice. For (hCB)-CD34^+^ cell propagation, BM cells were extracted by crushing bones from posterior limbs and sternum of already humanized NSG recipients. Total BM cells were then re-transplanted in sub-lethally irradiated (1 Gy) NSG recipients. Engraftment of (hCB)-CD34^+^ cells was controlled via flow cytometry using anti-human CD45 antibody (APC-conjugated, Miltenyi Biotec, Cat. No 130-091-230).

### Human AMLs

Two different primary human AMLs from IEO Biobank were used for transplantation in (hCB)-CD34^+^ NSG mice. hAML1 derived from a patient affected by an M4 AML, harbouring the t(9;11) translocation. hAML2 is normal karyotype leukemia, bearing mutations in NPM and FLT3 genes. These leukemias were xenotransplanted in NSG mice, and leukemic spleens of these mice were used in the experiments. 1x10^6^ leukemia cells per mouse were injected in the caudal vein of normal NSG or (hCB)-CD34^+^ NSG mice. Upon transplantation, mice were followed for disease development by controlling the haematological parameters (bleeding via tail vein was performed weekly). Mice have been also checked every 2-3 days to evaluate any sign of distress, such as pale extremities, curved posture, reluctance in motion. At the onset of the disease, mice were euthanized using CO_2_, and all efforts were made to minimize suffering.

### CD3/CD28 activation assay

96-well plate was coated with 5μg/ml of anti-human CD3 antibody (eBioscience, Cat. No 16-0037) for 2 hours at 37^o^C. Successively, cells were prepared and added to the plate (150.000 cells/well). Immediately after, 2μg/ml of anti-human CD28 antibody (eBioscience, Cat. No 16-0289) was added to each well and plate was incubated at 37^o^C for 4 days.

### CFSE proliferation assay

Human T cells, isolated from the spleens of (hCB)-CD34^+^ NSG mice, were stained using CellTrace^™^ CFSE Proliferation kit (ThermoFisher, Cat. No C34554), according to manufacturer’s protocol. Four days post culture, proliferation of these cells was assessed via FACS.

## Results and discussion

**Fig 1:**
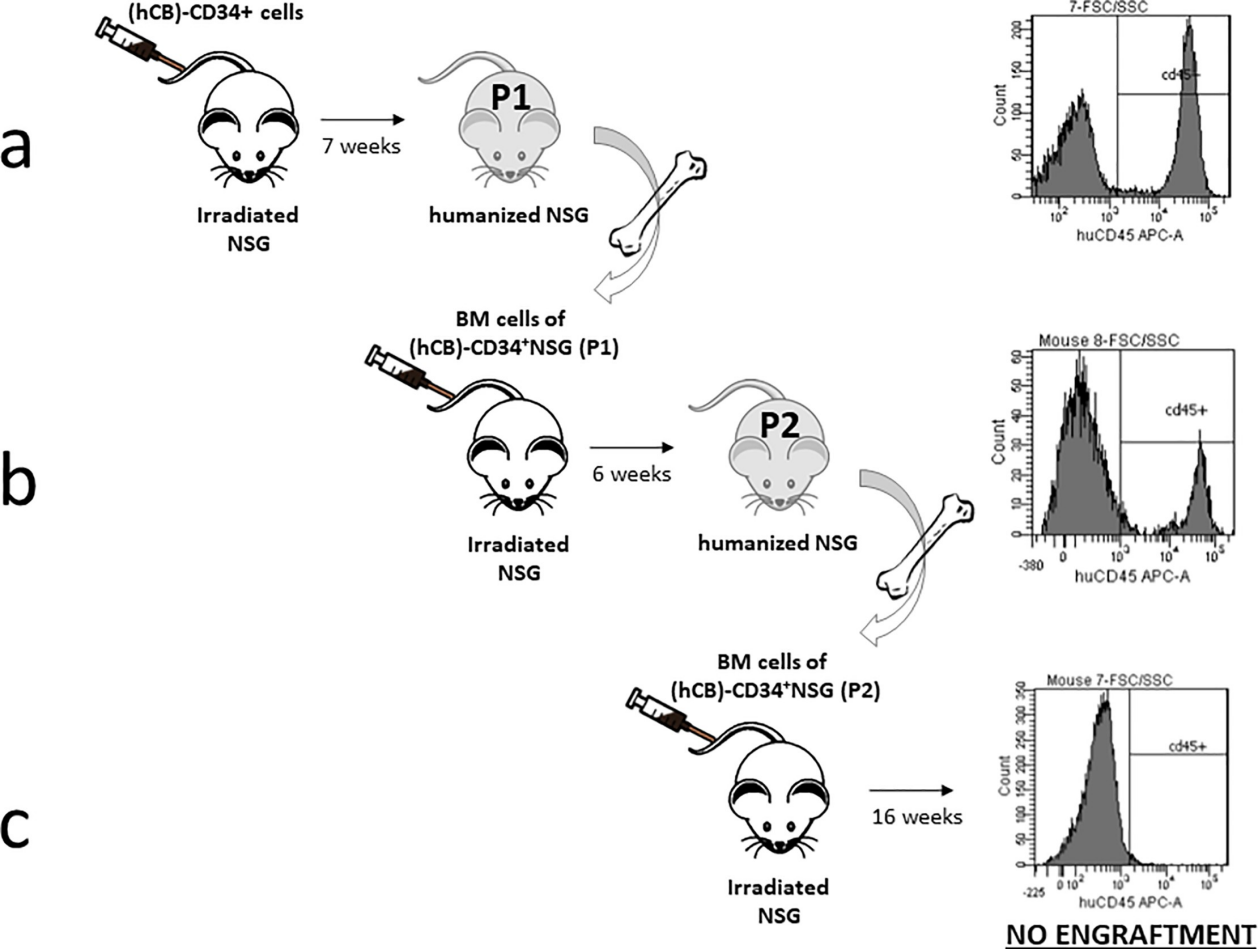
Generation of (hCB)-CD34^+^ NSG mice and propagation of (hCB)-CD34^+^ cells. NSG recipient mice (n = 9) were irradiated (1 Gy) prior to transplantation. Engraftment of human cells has been evaluated via detection of human CD45^+^ cells in the PB of (hCB)-CD34^+^ humanized NSG mice (flow cytometry analysis) a) engraftment at 7 weeks post-transplant of (hCB)-CD34^+^ cells in 3/9 transplanted mice (percentage of detected hCD45^+^ cells is 29.2±13.1) b) engraftment at 6 weeks post-transplant of BM from P1 (hCB)-CD34^+^ humanized NSG mice in 3/3 transplanted mice (percentage of detected hCD45^+^ cells is 18.7±2.3) c) engraftment at 16 weeks post-transplant of BM from P2 (hCB)-CD34^+^ humanized NSG mice in 0/4 transplanted mice (hCD45^+^ cells were not detected in any of the transplanted mice).

In order to study the interactions between human acute myeloid leukemia (hAML) and human immune system, we developed a humanized NSG mouse model by transplanting human CD34^+^ ((hCB)-CD34^+^) cells from the cord blood of healthy donors. Despite the fact that these mice develop impaired human T-cell compartment, due to the lack of human thymus[6], we aimed to test whether the partial inefficiency of the human immune system in this model could reduce a potential GvH response and allow human tumor growth. Finding a murine humanized system where the incomplete immunological response allows, to a certain degree, tumor growth would be, anyhow, a valuable tool for immuno-oncological studies.

(hCB)-CD34^+^ humanized NSG mice were obtained by transplanting human CD34^+^ cells from the cord blood of healthy donors into sub-lethally irradiated NSG mice. Seven weeks after transplantation, the percentage of CD45^+^ cells in the peripheral blood (PB) of transplanted mice was higher than 20% (measured via flow cytometry) (Fig 1A). Therefore, (hCB)-CD34^+^ cells engrafted and mice were considered humanized.

In order to amplify (hCB)-CD34^+^ cells and propagate (hCB)-CD34^+^ humanized NSG mice, the whole bone marrow (BM) of (hCB)-CD34^+^ humanized NSG mice was intravenously injected into irradiated NSG recipients (Fig 1B). The engraftment of (hCB)-CD34^+^ cells was detected already six weeks post transplantation. This may be due to stimulated self-renewal capability of (hCB)-CD34^+^ cells upon re-transplantation. Self-renewal is a typical feature of stem cells and it is a special process of cell division aimed to preserve the undifferentiated state of the stem cell[15]. However, it is an intrinsically restricted process and stem cells functionally exhaust after several rounds of division[15]. On the other hand, NSG mice transplanted with BM of second passage (hCB)-CD34^+^ humanized NSG mice did not show any sign of engraftment even 16 weeks post injection (Fig 1C). These observations might be explained by the fact that (hCB)-CD34^+^ cells are pushed to proliferate and rapidly undergo functional exhaustion in this model system.

**Fig 2:**
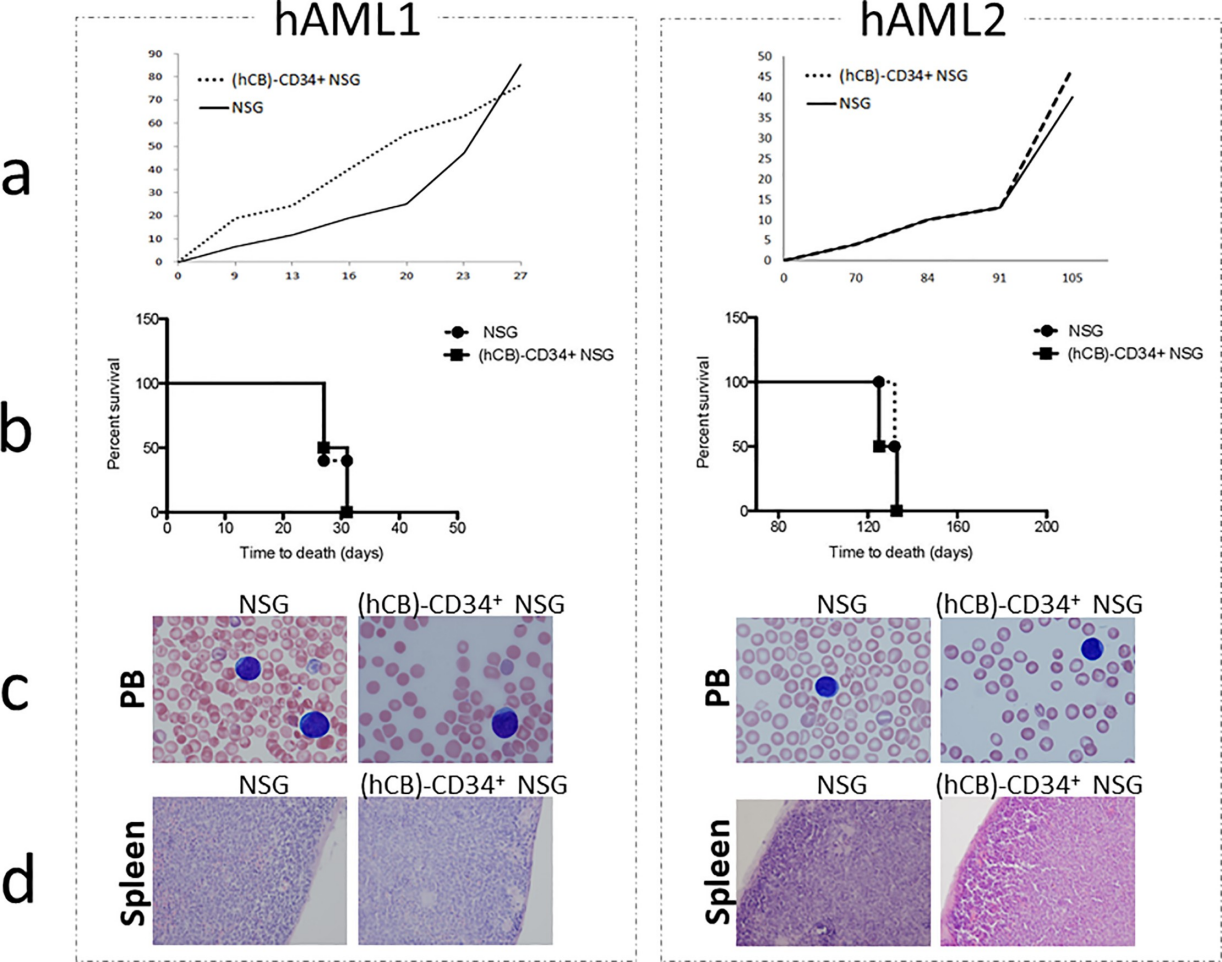
hAML grows in (hCB)-CD34^+^ NSG recipients. Transplantability of two different hAMLs (hAML1 and hAML2) in (hCB)-CD34^+^ humanized NSG and control NSG mice (at least two mice *per* group *per* each leukemia). a) kinetics of disease development represented as percentage of blasts in the PB. b) survival curve of humanized NSG *vs* control NSG mice transplanted with hAML1 and hAML2. c) leukemic blasts in blood smears of transplanted animals d) spleen architecture of transplanted animals.

We then investigated the accessibility of (hCB)-CD34^+^ humanized NSG mice to tumour growth, following the development of the disease in (hCB)-CD34^+^ humanized NSG mice upon human acute myeloid leukemia (hAML) transplantation. Leukemias from two AML patients have been intravenously injected in (hCB)-CD34^+^ NSG mice. Both control and (hCB)-CD34^+^ NSG mice transplanted with hAML developed leukemia with comparable timing and kinetics (Fig 2A and 2B). The features of leukemia, in terms of morphology of the blasts and organ infiltration by leukemic cells, did not show any difference between the two groups (Fig 2C and 2D). These data suggest a complete accessibility of the (hCB)-CD34^+^ NSG mice to leukemia growth, most likely due to an inefficient recognition of the human leukemia by the human immune components in the humanized context.

**Fig 3:**
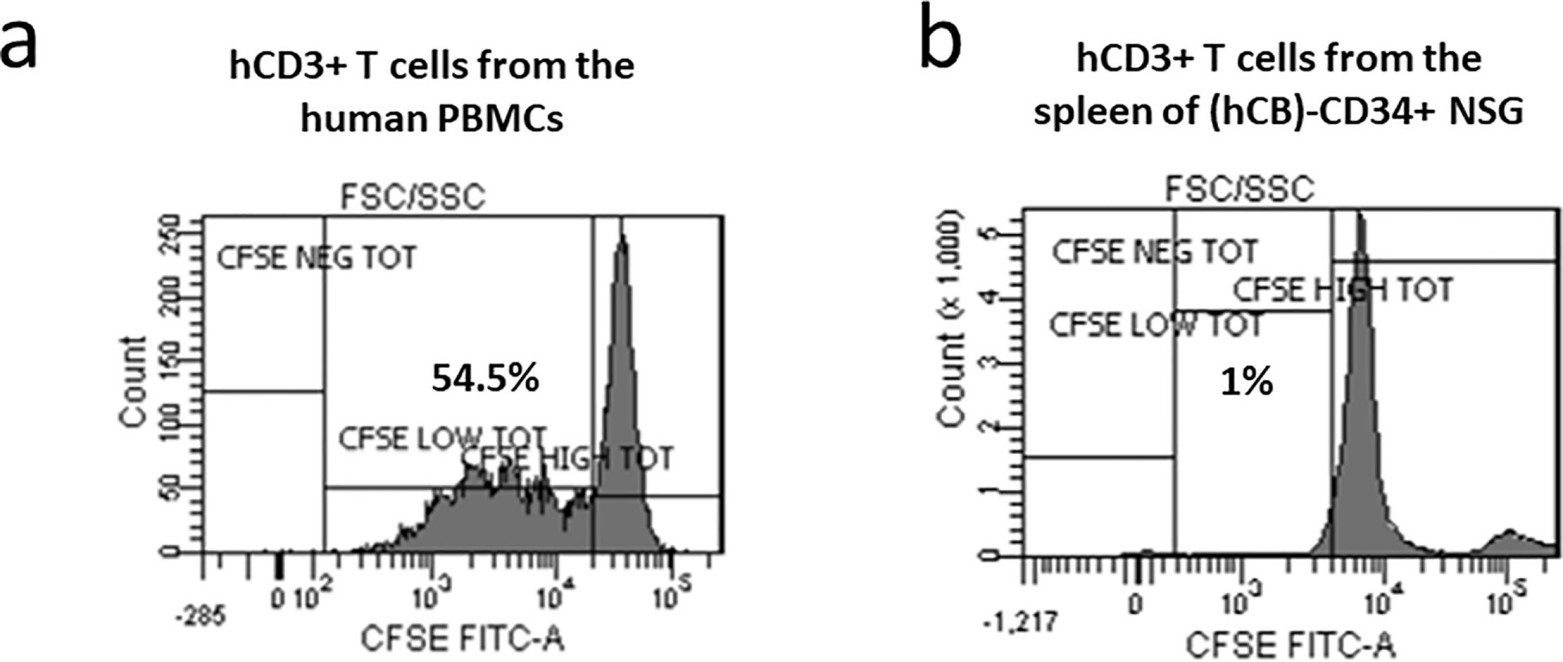
Human T cells developed in (hCB)-CD34^+^ NSG do not proliferate upon CD3/CD28 activation. FACS plots of CFSE-stained CD3^+^ human T cells isolated from a) human PB mononuclear cells as control and b) spleen of (hCB)-CD34^+^ NSG upon *in vitro* CD3/CD28 activation. Proliferating T cells are labelled as CD3^+^ CFSE^low^ cells.

Flow cytometry analysis of PB of (hCB)-CD34^+^ NSG showed the presence of all the major groups of human immune cell types, with high occupancy of naïve T cells, confirming that the (hCB)-CD34^+^ NSG mouse model develops a “primitive” human immune system. As reported by Watanabe Y. et al., *in vitro* CD3/CD28-stimulated human T cells derived from humanized mice do not produce cytokines (i.e. IL-2, INFN**γ**, IL-4), suggesting that these cells are not activated[11]. Accordingly, our *in vitro* CD3/CD28 activation along with CFSE assay (to easily quantify proliferating cells) demonstrated that human T cells from spleen of (hCB)-CD34^+^ NSG do not proliferate upon stimulation, confirming the development of entirely non-functional T compartment in our mouse model (Fig 3B). Human T cells isolated from the human PBMC have been used as positive control for the proliferation assay, since it is known that these cells do proliferate after a canonical CD3/CD28 activation (Fig 3A). The level of CFSE staining between human T cells isolated from humanized mouse spleen and human PBMC was not identical due to the different efficiency of CFSE staining of two cell populations. The “two populations” highlighted by the CFSE staining in Fig 3B are CFSE-stained human T cells (left, higher peak) and background contaminating cells from the mouse spleen (right, lower peak).

Our observations suggest that (hCB)-CD34^+^ NSG mice are transient and incomplete carriers of the human immune system and thus an insufficient model to study interactions between human immune system and human cancer. Despite the fact that this model develops all the components of human immunity, we did not find the conditions enabling cancer growth, where there is a balance between the response of the immune system and escape of the tumor. Actually, the (hCB)-CD34^+^ NSG is a fully accessible system to human tumor growth, comparable to a Xenograft model in the absence of the human immune system, and can be definitively abandoned as tool for translational studies of leukemia immuno-treatments. Therefore, further improvements are needed in order to achieve an ultimate humanized mouse model suitable for preclinical studies of human biology in the absence of an HLA (histocompatibility leukocyte antigen) correspondence between immunity and cancer. Importantly, it is not easy to obtain models where the immunity and the tumor components have a perfect HLA match. Therefore, it is craved the development of a humanized mouse model that offers an acceptable window of response/escape, amenable to study interactions between the human immune system and human cancer in not-fully-compatible conditions.
